# Ginger Bioactives: A Comprehensive Review of Health Benefits and Potential Food Applications

**DOI:** 10.3390/antiox12112015

**Published:** 2023-11-18

**Authors:** Muhammad Nouman Shaukat, Akmal Nazir, Biagio Fallico

**Affiliations:** 1 Department of Agriculture, Food and Environment, University of Catania, Via S. Sofia 100, 95123 Catania, Italy; nouman.shaukat@phd.unict.it; 2 Department of Food Science, College of Agriculture and Veterinary Medicine, United Arab Emirates University, Al Ain P.O. Box 15551, United Arab Emirates; akmal.nazir@uaeu.ac.ae

**Keywords:** ginger, bioactives, antioxidants, gingerols, health benefits, food applications, bakery, dairy, meat

## Abstract

Ginger is an herbaceous and flowering plant renowned for its rhizome, which is widely employed as both a spice and an herb. Since ancient times, ginger has been consumed in folk medicine and traditional cuisines for its favorable health effects. Different in vitro and in vivo studies have disclosed the advantageous physiological aspects of ginger, primarily due to its antioxidant, anti-inflammatory, antimicrobial, and anti-carcinogenic properties. These health-promoting features are linked to the variety of bioactive compounds that are present in ginger. Following the advancement in consumer awareness and the industrial demand for organic antioxidants and functional ingredients, the application of ginger and its derivatives has been broadly investigated in a wide range of food products. The prominent features transmitted by ginger into different food areas are antioxidant and nutraceutical values (bakery); flavor, acceptability, and techno-functional characteristics (dairy); hedonic and antimicrobial properties (beverages); oxidative stability, tenderization, and sensorial attributes (meat); and shelf life and sensorial properties (film, coating, and packaging). This review is focused on providing a comprehensive overview of the tendencies in the application of ginger and its derivatives in the food industry and concurrently briefly discusses the beneficial aspects and processing of ginger.

## 1. Introduction

Ginger (*Zingiber officinale* Roscoe) plant, producing an irregular bumpy rhizome, is extensively cultivated in the southeast region of Asia. Accurate information regarding ginger plant’s origin is not available due to a very long cultivation history in this region. In ancient Greek history (40–90 AD), the Greeks were familiar to the dietary and culinary uses of ginger. In the 13th century, during his visit to China and Sumatra, Marco Polo became acquainted with ginger and imported it to Europe. In the same era, the Arabs transported the ginger from India to East Africa, while the Portuguese spread it to West Africa [1]. 

It is an important dietary ingredient in daily South Asian cuisine and traditional Chinese medicine. In the entire history, ginger rhizome, commonly known simply as ginger, is not merely utilized as a spice or herb to impart various food attributes like flavor, aroma, food preservation, and nutritional reform, but it has also been administered for the mitigation of various ailments such as asthma, flu, indigestion, and gastrointestinal discomfort [2]. 

Tropical and subtropical climates are favorable for ginger cultivation because it cannot combat very low temperatures. When ginger is utilized as a vegetable or for the production of ginger preserve, pickles, candies, and beverages, the recommended harvesting time is about 4–5 months after plantation, whereas when its intended employment is dried ginger and for the manufacturing of bleached and dehydrated ginger or functional products like ginger oil and oleoresin, the recommended harvesting period is around 8–10 months [3]. The height of a ginger plant is usually around 2 feet with yellow bloomed flowers characterized by a pungent smell. The underground stem or rhizome of ginger, attributed with a hot and strong spicy aroma flavor, used for medicinal and culinary purposes, is also one of the most abundantly used spices belonging to the *Zingiberaceae* family [3,4]. The ginger plant has a very close relevancy to other herbal plants like cardamom (*Elettaria cardamomum*) and turmeric (*Curcuma longa*) as they are members of the same family (*Zingiberaceae*). Ginger comprises carbohydrates (60–70%), water (9–12%), proteins (9%), lipids (3–6%), ash (8%), crude fiber (3–8%), and essential oil (2–3%). Apart from these macronutrients, ginger also contains various bioactive compounds and some mineral elements like sodium, potassium, calcium, magnesium, and phosphorus [5]. There is also a possibility of synthesizing new compounds when there is any alteration in the ginger’s growth cycles, for example, during the storage of ginger. The time period, humidity level, and temperature are also the influencing factors of the normal composition of ginger [6]. The presence of ketones in the chemical composition bestows the unique spicy aroma to these plants [7]. 

Ginger has been the subject of extensive research in the past owing to its possible health advantages. Several authors have reviewed the available literature and discussed the bioactive compounds present in ginger, the techniques used for their extraction, health promoting features, the encapsulation of ginger bioactives, and some specific or brief food applications of ginger and its derivatives [6,8,9,10,11,12,13]. However, there is a lack of reviews regarding the application of ginger to different food categories. It is also imperatively important to mention that the primary objective of incorporating ginger and its derivatives into a food product is to improve the nutraceutical value and shelf life of the relevant product behind which the lead character, directly or indirectly, belongs to the antioxidant compounds and their antioxidant activities. Therefore, the present paper is designed to furnish a comprehensive review of ginger application in different food sectors, while also briefly discussing the bioactive components, the effect of processing and extraction methods, and potential health benefits.

## 2. Bioactive Compounds in Ginger

**Figure 1:**
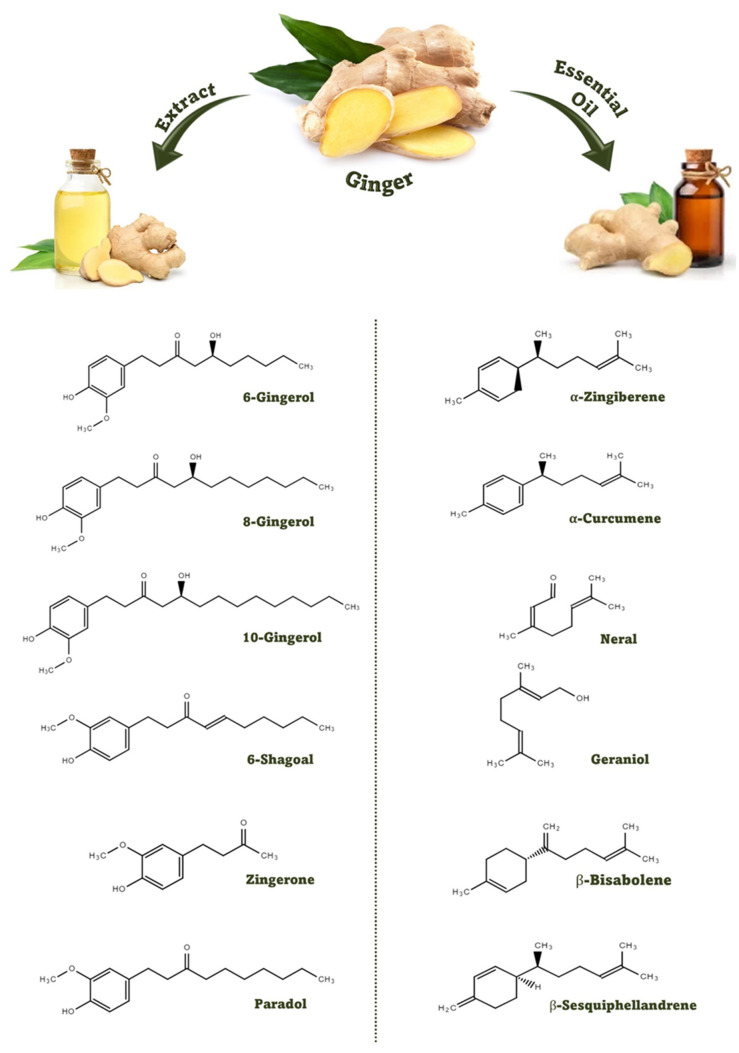
Chemical structures of major bioactive compounds present in ginger.

The nutritional and nutraceutical benefits of ginger are closely associated with the presence of various bioactive compounds. These bioactive compounds, reported in different studies, are classified into three classes: gingerols, volatile oils, and diarylheptanoids [14]. The chemical components of ginger include volatiles (geraniol, borneol, terpineol, zingiberenol, cineole, limonene, camphene, curcumene, zingiberol, geranyl acetate, linalool, α-Farnesene, α-sesquiphellandrene, α-zingiberene, β-bisabolene, β-elemene, and β-phellandrene) and non-volatiles (gingerols, shogaols, zingerone, and paradols). These non-volatile phytochemicals, having pungent odors and flavors, are the characteristic bioactive constituents of ginger. The distinctive aroma flavor of ginger is the resultant blend of these three non-volatile active compounds (gingerol, shogaol, and zingerone), which constitute one to three percent of fresh ginger by weight. There are two common components, vanillyl (4-hydroxy-3-methoxphenyl) and the ketone functional group, that are found in every pungent compound of ginger [7,15]. The chemical structures of the major bioactive compounds that are present in ginger extract and essential oil are presented in Figure 1.

Gingerol is a pungent oil, yellow in color, that is also found in crystalline solid form with a low melting point. Gingerols are classified into 4-, 6-, 8-, 10-, and 12-gingerol based on the length of the unbranched alkaline chain. 6-Gingerol is the most abundantly available type of gingerol, while considerable amounts of 8-gingerol and 10-gingerol are also found in fresh ginger [16,17]. 6-Gingerol is a moderately pungent and vital active compound of ginger with anti-inflammatory, anti-bacterial, and anti-carcinogenic properties. 6-Gingerol is reported to have different health-beneficial properties like anti-carcinogenic, antioxidant, anti-inflammatory, cardiotonic and hypotensive, anti-emetic, anti-pyretic, anti-rheumatic, anti-ulcer, anti-prostaglandin, and many other properties. 8-Gingerol is the most pungent and one of the most active phytochemicals in ginger. It has been cited with many health benefits by various researchers [18,19]. 

Just like the 6-gingerol in gingerols, 6-shogaol is the most amply available and most effective type of shogaol in ginger. 6-Shogaol is also described with many pharmacological and health characteristics like anti-inflammatory, anti-ulcer, anti-prostaglandin, anti-carcinogenic, anti-invasive, and many other properties. 6-Shogaol has a very powerful expectorant or anti-tissue effect, which may assist in reducing blood pressure. Some studies also disclosed that 6-shogaol, the dehydration product of 6-giongerol, is probably more functionally active than its precursor [20,21,22]. Zingerone, also known as vanillyl acetone, is a bioactive compound of ginger that is available in crystalline solid form. Its chemical makeup is very similar to other flavor compounds like eugenol and vanillin [23]. 

The bioactive composition of ginger essential oil, mainly comprising the volatile oils of ginger, is different than ginger extract. The functional compounds of ginger essential oil are classified into two main groups: monoterpenes and sesquiterpene hydrocarbons. The chemical composition of ginger essential oil is also affected by the nature (fresh or dried) and source of ginger rhizome [5]. The most abundantly available bioactive compounds of ginger essential oil are *α*-curcumene, *α*-zingiberene, geranial, β-sesquiphellandrene, β-bisabolene, and neral. Ginger essential oil has also been characterized by a diversified extent of bioactivity and health-promoting properties [24]. 

## 3. Effect of Processing and Extraction Process on Ginger Bioactive Compounds

Due to its high moisture (ranging from 85–95%), fresh ginger rhizome is susceptible to spoilage and decay. During storage, the time and temperature have remarkable impacts on the bioactivity, phytochemical profile, and overall quality of ginger rhizome. To avoid postharvest losses and to maintain the quality of fresh food commodities, different processing and preservation techniques such as drying, salting, canning, freezing, vacuum packaging, etc., are practiced in the food industry [25]. Among these processing technologies, different drying methods with a lot of variations and advancements have been employed for ginger processing. Apart from a longer shelf life, dried ginger is also superior to fresh ginger because of its better compatibility and yield for the extraction of bioactive compounds. The advanced and latest drying technologies including freeze-drying and infrared heating are advantageous over conventional drying methods but confined due to some limitations (such as the processing time, higher energy demand, and expensive equipment). A supreme quality product could also be obtained by optimizing the processing parameters, like the temperature and time, under controlled climate conditions [26]. 

**Figure 2:**
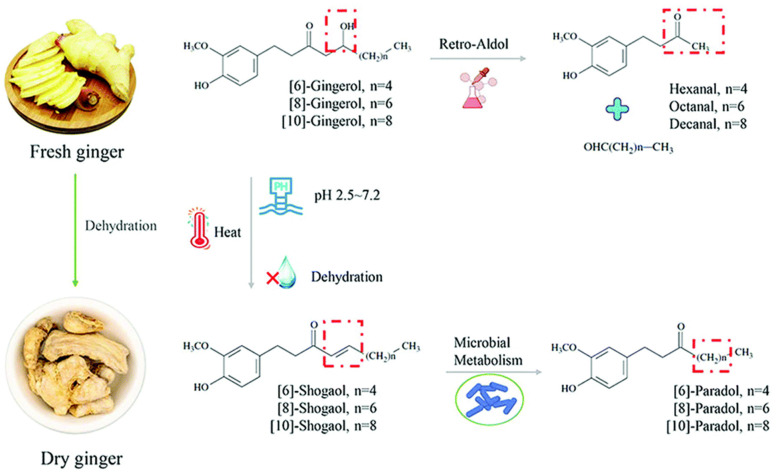
Effect of processing on ginger bioactives and its constituents [6].

The selection of an appropriate processing technique along with optimum processing parameters is essential to obtain the desired end product. The processing conditions could lead towards changes in the phytochemical content, bioactive profile, and overall makeup of ginger (Figure 2) [27]. For instance, during drying, more pungent compounds, i.e., shogaols, are produced during the dehydration process. Zingerone, a mild spicy and pungent component with a sweet aroma, is derived during the cooking of ginger. Gingerols are destabilized during the thermal or drying process, and also during storage, which results in a mutation at the C-5 of gingerol through the removal of the OH group. The removal of this OH group results in the production of a double bond between the carbon positions of C-4 and C-5. Therefore, shogaols can be produced from the dehydration of heat-unstable β-hydroxyl ketone and gingerdiones to form an α, β-unsaturated ketone [28]. 

Gingerols are sensitive to some processing and storage conditions like thermal processing, acidic environments, air, light, and prolonged storage. When gingerols undergo these conditions, there is a possibility of the transformation of gingerols into shogaols [29]. Studies also revealed that during the dehydration process of ginger, the quantity of 6-gingerol decreased with the increase in 6-shogaol [30]. Moreover, the microbial metabolism of shogaol generated the β-ketone hydroxyl deoxygenated group of products named paradols. During the heating of fresh ginger, zingerone is formed from the gingerols through a retro aldol reaction [23]. 

The chemical composition and yield of ginger extract and ginger essential oil are also influenced by the extraction method. Advanced and green extraction techniques are preferred over classical extraction techniques [11]. The extraction techniques with low temperature requirements such as supercritical fluid extractions, ultrasound-assisted extractions, and enzyme-assisted extractions are usually employed to avoid the degradation or conversion of heat-sensitive bioactive compounds. The extracts acquired from these extraction processes are relatively rich in gingerols, antioxidants, and total phenols. Meanwhile, microwave-assisted extraction, pressurized liquid extraction, and acidic solvent extraction are useful to obtain 6-shogaol-rich extracts due to the possible transformation of 6-gingerol to 6-shogaol under stress conditions. Microwave-assisted and ultrasound-assisted extraction render an economical edge of being time-efficient, while supercritical fluid extraction is acknowledged for the safety and quality of the product. [9,10]. If a comparison is drawn on the basis of the phenolic profile, conventional solvent extraction using water and ethanol as solvents resulted in total phenolic contents of 104.7 ± 4.5 mg GAE/100 g and 650.44 ± 27.32 mg GAE/100 g of dry weight, respectively [31,32], while the extract of freeze-dried ginger powder produced through sonication came up with a total phenolic content of 931.94 ± 0.02 mg GAE/100 g of dry weight [27].

Furthermore, a synergistic effect with a superior quality product could also be achieved through the consolidation of these extraction techniques, and this is the most emerging approach in the field of extraction to maximize the extraction efficiency through the synchronization of two or more advanced extraction technologies [33]. For instance, the efficacy of using different natural deep eutectic solvents (NADESs) as green extraction solvents has also been evaluated in combination with ultrasound-assisted extraction. This consolidation has emerged successfully with favorable results as the total phenolic content of the extract obtained from this venture was 2010 ± 26 mg GAE/100 g of dried ginger powder, which is remarkably higher than the previously described extraction techniques [34]. 

## 4. Beneficial Aspects of Bioactive Compounds

As mentioned above, the functional and bioactive compounds present in ginger are associated with multiple health benefits and advantageous characteristics. The exploitation and characterization of the versatile biological and functional competencies of ginger are also crucial for further research. These multifaceted bioactivities of ginger bioactives and their modes of action are discussed here in this section.

### 4.1. Antioxidant Activity

Any disturbance or adjustment with the cell’s antioxidant defense system can lead to the excessive production of Reactive Oxygen Species (ROS), and eventually, to an increase in oxidative stress. For the recovery of normal levels of free radicals and oxidative stress, various natural antioxidants have been analyzed for the neutralization of free radicals and encounter oxidative stress for the prevention of different health disorders [35]. The maintenance of a normal ROS level in a living organism is very important for normal functioning, which can be achieved through the provision of hydrogen atoms or electrons to free radicals to hinder the ROS production and convert them into antioxidant lipid complexes or thermally stable products [36]. 

In the past two decades, the exploration of natural plant resources has become very attractive and beneficial, most probably due to the presence of bioactives, less toxicity, and the lower production costs [37]. Ginger bioactive compounds have significant antioxidant potential to restore or maintain normal oxidative stress levels. Ginger leaves were reported to have more antioxidative potential than ginger rhizome and ginger flowers [32,38]. The higher in vitro antioxidant activity (88.93 ± 0.03% DPPH; 88.23 ± 0.98% ABTS) was noted in 6-gingerol. The daily oral administration of 6-gingerol (50–75 mg/Kg body weight) to mice for three weeks enhanced their blood glucose levels, reduced their oxidative stress, and also improved their insulin signaling. A higher antioxidant activity of shogaols was recorded as 89.01 ± 0.6% and 90.2 ± 0.11% for ABTS and DPPH, respectively [39,40]. The highest antioxidant potential of 6-shogaol compared to other phenols of ginger is due to the existence of a special functional group (α, β-unsaturated carbonyl), which is efficiently involved in the regulation of glutathione. Glutathione is the most amply available type of thiol in animal cells, and it is engaged in the cell detoxification mechanism against detrimental substances and intracellular redox management [41]. 

### 4.2. Anti-Inflammatory Activity

Inflammation is a phase in the natural healing process of the body to combat intruders for the prevention of infection or injury. The basic aim of inflammation is to abrogate cellular damage, withdraw and absorb necrotic cells and tissues, and regain the stability and conformity of the intracellular environment [42]. When cells go through severe inflammation, a large number of mononuclear immune cells are penetrated, which stimulates the production of inflammatory interleukins, cytokines, and tumor necrosis factors including inflammation-promoting cytokines, such as tumor necrosis factor α (TNF-α), interleukin-1β (IL-1β), and interleukin-6 (IL-6), which are very significant for the inflammation process. A recent study focused on the anti-inflammatory characteristics of ginger mentioned that ginger consumption could substantially lower the TNF-α serum levels but has a minor impact on IL-6 [43]. 

It was also observed that the anti-neuroinflammatory capacity of gingerols enhanced with the increase in the alkyl chain length as 10-gingerol’s anti-neuroinflammatory activity was higher than the other active compounds of ginger [44]. Furthermore, different in vivo studies have also supported these claims through the practical implication of ginger bioactives. Nanoparticles derived from ginger that were orally administered to mice, having acute and chronic colitis, at the dosage rate of 0.3mg/mice on a daily basis for one week efficiently halted intestinal inflammation by decreasing the pro-inflammatory cytokines like TNF-α, IL-1β, and IL-6 and increasing the levels of anti-inflammatory cytokines like IL-10 (Interleukin-10) and IL-22 (Interleukin-22). In mice suffering with dextran sulfate sodium-induced colitis, when 6-shogaol-equipped nanoparticles (equivalent to 15 mg/kg of 6-shogaol) were orally administered on a daily basis for a week, they not only diminished the colitis symptoms but also boosted the healing process [45,46].

### 4.3. Antimicrobial Activity

Currently, chemical and synthetic antimicrobials are being replaced with natural and organic antimicrobials derived from plants, animals, and microorganisms, both in health management and in food preservation. These biologically derived antimicrobials from plant sources are preferred due to their multi-oriental management, multi-targeted approach, drug resistance, and lower toxicity [47]. Ginger and its derivatives have been appraised for their tremendous anti-bacterial, anti-viral, and anti-fungal activities [48]. Microorganisms tend to make biofilm as their strong defense against antimicrobials. A study carried out by Chakotiya et al. [49] reported that ginger has shown an inhibitory action against *Psedomonas aeruginosa* (a multi-drug-resistant strain) by disturbing the membrane stability and preventing biofilm formation. 

Ginger essential oil possesses excellent anti-bacterial activity against food-borne pathogenic bacteria such as *Escherichia coli* and *Staphylococcus aureus*. A study reported the bactericidal mechanism of ginger essential oil through the demolishment of the cell membrane activity and through intervening with the energy metabolism because of some macromolecular elements like proteins and nucleic acids [50]. Another study expressed that fresh ginger oil had a moderate antimicrobial effect on *Candida*, *Aspergillus niger*, and *P. aeruginosa* and a mild effect against *Saccharomyces cerevisiae*, and no inhibition was detected against *Bacillus subtilis*, *Trichoderma* spp., and *Pencillium* spp. Ginger oil obtained from dried ginger was observed to have higher anti-bacterial activity against *P. aeruginosa* [51]. There is a close correlation between anti-bacterial and antioxidant properties as the blocking of antioxidant activity could result in the detention of anti-bacterial activity. Processing and storage conditions could also be made changes with functional attributes of ginger resulting in difference in antioxidant and anti-bacterial activities [52].

### 4.4. Anti-Carcinogenic Potential

Cancer is one of the major causes of death in the world, and in 2020, approximately 19 million cancer cases were reported, out of which about 10 million cases worldwide were fatal [53]. In recent years, ginger has been extensively investigated for its anti-carcinogenic activities against different types of cancer, like cervical, breast, colorectal, and prostate cancers [54,55]. Chronic inflammation plays a key function in carcinogenesis as it has a close connection with all crucial stages of cancer formation [56]. Ginger and its active compounds have been identified with anti-carcinogenic properties against all stages of cancer development including cancer initiation, promotion, progression, and drug resistance. An in vitro study disclosed that a fraction loaded with polyphenols derived from dried ginger powder restrained the progression of gastric adenocarcinoma cells and colorectal cancer cells [57].

In vivo and in vitro studies were carried out to evaluate the action mechanism and cytotoxic effects of ginger against prostate cancer [58]. It was reported that 6-gingerol, 6-shogaol, 10-gingerol, and 10-shogaol showed considerable anti-proliferative effects on prostate cancer cells in humans through the deregulation of the protein expression of MRP1 (multi-drug resistance-associated protein 1) and GSTπ (Glutathione-S-transferase). The proliferation of PC-3 prostate cancer cells was synergistically inhibited through the bifold consolidation of ginger bioactives like 6-gingerol, 8-gingerol, 10-gingerol, and 6-shogaol [59]. 6-Gingerol stimulated the increase in ROS formation in human gastric adenocarcinoma (AGS) cells, which promote apoptosis and reduce the mitochondrial membrane potential [60]. The in vitro application of 6-shogaol (10–20 μM) to PC12 cells for 24 h improvised the phase II antioxidant compounds at the pheochromocytoma cell line in mice, which effectively inhibited the proliferation of oxidative stress in PC12 cells. On the contrary, 6-gingerol failed to protect the PC12 cells from the oxidative stress, possibly due to the absence of an α, β-unsaturated ketone, which is an indicator of the α, β-unsaturated ketone unit’s importance in cytoprotection [61].

### 4.5. Neuroprotective Activity

There is a possibility of an onset of neurodegenerative diseases such as Alzheimer’s disease and Parkinson’s disease, and old people are at an especially high risk. Many recent studies have expressed that ginger possesses anti-neuroinflammatory capacity and has a significant role in memory function, which might be useful in the management and inhibition of neurodegenerative diseases [62,63]. It was also disclosed that 10-gingerol from fresh ginger exhibits powerful anti-neuroinflammatory activity, proven through a lipopolysaccharide-activated BV2 microglia culture model. 10-Gingerol restricted the articulation of pro-inflammatory genes by inhibiting NF-κB activation, which ultimately led to a decrease in the levels of IL-6, IL-1β, NO, and TNF-α. [44]. The phytochemicals derived from ginger such as gingerols, shogaols, and others, at a concentration of 20 μM, have been reported with strong neuroprotection capacities, especially against Alzheimer’s disease. Ginger could lower the Aβ-species and cerebral plaques, inhibit the cerebral inflammation, amend the tau protein, and restrain Aβ-induced apoptosis through different pathways [64]. 

### 4.6. Cardiovascular Protection

Worldwide, the dominant cause of premature deaths (17.9 million people per year) is cardiovascular disease [65]. Various studies revealed that ginger can efficiently provide protection against cardiovascular diseases by reducing the levels of blood pressure and blood lipids. The level of high-density lipoprotein cholesterol (HDL-C), also known as good cholesterol due to its defensive stature against cardiovascular diseases, increased in the serum, while a decrease in body weight was observed in mice on a high-fat diet supplemented with 0.5% of lyophilized ginger extract for a period of about two weeks. Ginger extract also facilitated high-density lipoprotein (HDL) production through enhancing the levels of lecithin–cholesterol acyltransferase RNA and apoliprotein A-1 in the liver. Another study also supported the role of ginger extract in the reduction in low-density lipoprotein (LDL) and total cholesterol, and the integrated daily oral application of aqueous ginger extract (250 mg/kg of feed) for about a month and aerobic exercise increased the HDL levels in high-fat-diet-fed rats [66,67]. 

### 4.7. Anti-Obese Activity 

Obesity or overweight is a great concern throughout the world, and it emerges from the disturbance in the energy metabolism. Basically, there are two types of adipose (fat) tissues classified on the basis of their arrangements and activities. The core function of white adipose tissues is to serve as energy depositories, while brown adipose tissues are responsible for metabolism improvement and energy expenditure. The transformation of white adipose tissues to brown adipose tissues is a novel approach to tackle obesity. 6-Gingerol could stimulate the cell browning via an AMPK-dependent approach in 3T3-L1 adipocytes [68,69]. Gingerenone A was discovered as a better performer than gingerol and shogaol with efficient inhibition activity against adipogenesis and fat accumulation in 3T3-L1 preadipocyte cells. In this study, the oral administration of Gingerenone A (50 mg/kg of bodyweight) on a daily basis for 15 weeks also influenced the fatty acid metabolism through the in vivo activation of AMPK, debilitating diet-induced obesity [70].

### 4.8. Anti-Diabetic Effect

Diabetes mellitus is a chronic metabolic disease characterized by the insufficiency of insulin and/or insulin resistance, resulting in an uncontrolled increase in the blood glucose level. Persistent hyperglycemia could promote protein glycation and the generation of advanced glycation end products (AGEs). An in vitro study revealed that 6-gingerol and 6-shogaol inhibited the proliferation of diabetic complexities and confined the methylglycoxal (MGO), the precursor of AGEs, to hinder AGE production [71]. The oral supplementation of 6-gingerol (200 mg/kg of corn oil) on a day-to-day basis for 28 days to type 2 diabetic mice stimulated glycogen synthase 1 and improved the GLUT4 (glucose transporter type 4) cell membrane presentation, which raised the glycogen storage levels in the skeletal muscles [72]. 

## 5. Food Applications

**Figure 3:**
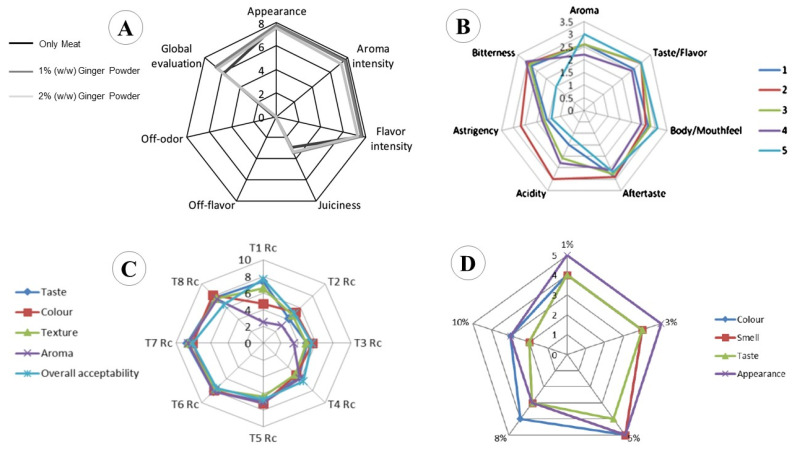
Sensory evaluation of different food products with incorporated ginger. (**A**) Pork burgers with added ginger powder [137]. (**B**) Comparison of ginger-based beverage with common coffee types. 1—traditional coffee; 2—ginger-based beverage; 3—instant coffee; 4—espresso coffee; 5—American/filter coffee [134]. (**C**) Different formulations of ginger-supplemented cookies. Ginger powder with concentrations of 0, 2, 4, 6, 8, 10, 12, and 14% for T1 to T8, respectively [82]. (**D**) Cottage cheese supplemented with natural ginger extract at a concentration of 1% to 10% of the total volume of the cottage cheese [158].

Currently, there is a growing trend towards healthier and functional food products as consumers are more conscious about healthy lifestyles and daily intakes. Due to prospering recognition and interest in functional food products, the scope for the exploration, characterization, and potential utilization of functional food ingredients and bioactive compounds has also magnified. Ginger, being a valuable functional ingredient and marvelous source of bioactives, has also been integrated into a variety of food products. Here, the food applications of ginger in its different forms (powder, juice, extract, and essential oil) will be discussed to provide a comprehensive and broader prospective of its employment. The impact of ginger incorporation on the sensorial attributes of various food products is also discussed below (Figure 3).

### 5.1. Bakery Products

A variety of studies have been carried out to use ginger as a potential food additive in bakery products (Table 1). For instance, the supplementation of ginger powder at a rate of 2–4% was reported, which can promise feasible textural, rheological, and antioxidant properties along with a better mineral content in bread [73]. Balestra et al. [74] formulated a functional bread using ginger with acceptable physicochemical and sensorial characteristics. They found that up to 3% of ginger powder can be incorporated into the bread without compromising the rheological properties of the dough and bread. Another study also came up with similar findings over the functional, physicochemical, and sensory properties of bread fortified with ginger powder but with a different optimized level of ginger powder (1%) than the previous study [75]. Moreover, Almasodi [76] studied the addition of ginger powder in bread as well as in rusk. They concluded that the incorporation of up to 3% and 6% of ginger powder resulted in satisfactory hedonic and textural properties for bread and rusk, respectively. In another study, garlic and ginger powders at different concentrations were also incorporated in wheat bread. Besides functionality improvement, it also increased the shelf life of the bread from 5 days to 7 days at an ambient temperature [77]. Ginger and turmeric have inhibitory effects on the growth of *Rhizopus stolonifer* on bread, but ginger may impart unfavorable flavor characteristics to the bread [78]. 

The aforementioned studies mainly dealt with the addition of ginger to produce functional bread. However, in some parts of the world, distinct baking products exist wherein ginger is used as a dominant flavoring ingredient. For instance, gingerbread is a traditional bakery product in the Czech Republic, particularly in the town of Pardubice, where ginger is the prevalent flavor. It is produced in a variety of shapes, types, and textures ranging from a crispy biscuit to a soft cake [79,80]. Hence, ginger has been practiced as a flavor enhancer, solely or in a combination, for a long time, but it is largely dependent on the nature of the food product, the form of the ginger to be incorporated, their mutual compatibility, and the sensorial preferences of the consumers. 

Lee et al. [81] proposed that functional cookies can be prepared with a 4% incorporation of ginger powder, as this concentration was found to have overall acceptability and a very bland effect on the quality characteristics. Kaushal et al. [82] also investigated the functional and sensorial properties of cookies with the addition of ginger powder. The functional and organoleptic properties of cookies supplemented with 12% ginger powder were very favorable compared to the controls. In another investigation, Yang et al. [83] also evaluated the effect of ginger substitution on the acrylamide content in dough and biscuit properties. Ground ginger addition at the rate of 1% was reported with favorable structural and color characteristics and acceptable sensorial characteristics with a 6.2% decrease in the acrylamide content. The substitution of ginger at higher rates decreased the acrylamide content, but the hedonic properties were not feasible. In principle, gingerols reduced the acrylamide content through inhibition where the phenol hydroxyl group of gingerol played a vital role. Kumari and Gupta [84] also utilized ginger powder and some other functional plant ingredients in the production of value-added cookies. Acceptable organoleptic characteristics were found with 2.5% of ginger powder. 

The functional, nutritional, antioxidant, and sensorial properties of millet flour and ginger powder-formulated cookies were investigated by Marak et al. [85]. This study reported that ginger flour incorporation of up to 10% was found satisfactory for the textural and sensorial properties of cookies, and it also efficiently improved the functional and nutritional values of cookies with a potential lead towards shelf life extension. When cumin and ginger were utilized for the preparation of cookies, a minimal difference was observed in the dough development as compared to the control [86]. Both potential ingredients were integrated in concentrations of up to 5% in soft wheat cookies, but there was not any substantial effect on the end product. Keeping in view the acceptability and feasibility indicators, these concentrations were also recommended for commercial scale production. The antimicrobial effect of ginger was also evaluated through the addition of ginger powder into biscuits. Aerobic bacteria were decreased proportionally by increasing the amount of ginger powder in biscuits. Functional biscuits with significant activity against bacteria and mold could be produced with around a 2.5% addition of ginger powder without any compromise on the quality characteristics [87]. Adebayo-Oyetoro et al. [88] also prepared ginger spiced cookies with around a 3% addition of ginger powder.

**Table 1:** Some important studies on the application of ginger and its derivatives to bakery products.

Derivative	Product	Positive Effect	Reference
Ginger Powder	Wheat Bread	↑ Antioxidant Activity; Minerals; Total Phenolics; Textural Properties; Shelf Life; Sensorial Characteristics; ↓ Microbial Growth	[73,77]
	Cookies	↑ Antioxidants; Phenolic Contents; Shelf Life; ↓ Acrylamide Content; Microbial Growth; ≈ Sensorial Characteristics	[82,83,85,86]
	Cake	↑ Antioxidant Activity; Total Phenolics, Shelf Life; Sensorial Properties; ↓ Bacterial and Fungal growth	[89]
Ginger Juice	Wheat Flour Muffin	↑ Total Phenolics; Antioxidants; Sensorial Characteristics; ≈ Textural Attributes	[90]
Ginger Extract	Baked Bars	↑ Antioxidants; Phenolic Contents; Antioxidant Retention during Storage; Flavor and Color; ≈ Overall Acceptability	[91]

↑ = Increased/Improved, ↓ = Lower/Decreased, ≈ = Negligible/Unchanged.

To a large extent, Almasodi [89] successfully incorporated ginger powder in butter cake and biscuits at the rates of 5% and 10%, respectively. Storage studies revealed that, apart from providing a nutritional boost, ginger powder also supplemented bakery products with natural antimicrobial compounds. In another recipe, the water content was substituted with ginger juice at three different concentrations from 10% to 30% to analyze the quality attributes of muffins [90]. Significant differences were observed in the color, texture, and taste with the increasing amount of ginger juice. Ginger juice at 10% of the water used in the recipe was recommended as the optimum amount for muffin production. The conventional and supercritical extracts of ginger were also analyzed through supplementation in baked bars at 3% and 0.3% concentrations, respectively [91]. The supercritical extract performed better than the conventional extract to improve and maintain the phenolic contents and antioxidant activities in storage duration. A sensorial evaluation also declared the supercritical ginger extract to be better than the control and conventional extract.

As per the foregoing studies, ginger powder is the most widely employed form of ginger in bakery products, followed by ginger juice. The pertinent amount of ginger powder and processing conditions are the key factors in the acquirement of balanced functional bakery items without any alteration in the desirable characteristics of the product. So, bakery products with admirable nutraceutical values could also be produced through the precise supplementation of ginger. 

### 5.2. Dairy Products

There is also a considerable potential for innovation and improvement in the dairy sector, where ginger, an active ingredient, has also contributed (Table 2). The supplementation of ginger powder (1%) has been displayed as a bovine yogurt product with an enhanced nutritional value, better quality characteristics, and ease in the production process [92]. Ginger powder is also formulated with dromedary milk to prepare and characterize a functional yogurt product. The resultant product showed very similar results to the previous study (bovine milk yogurt) with the recommendation of 1% ginger powder as an optimum supplementation extent. The water holding capacity, viability of the starter culture, and rate of pH reduction was increased, while the fermentation time and flow behavior was decreased, leading towards an acceptable yogurt from dromedary milk [93]. Ethiopian cottage cheese, ‘Ayib’, was also treated with ginger and garlic powders to analyze their impacts on the chemical arrangement and sensorial characteristics of the product [94]. Ayib treated with garlic powder was described with better overall acceptability than ginger powder, and further investigations were suggested to explore the biochemical and microbial properties of both powders.

A novel approach was also attempted to evaluate the effects of ginger, cinnamon, and carob powders on spray-dried milk powders and their agglomerates [95]. Milk powder with added ginger powder relatively performed better in terms of solubility, hygroscopicity, flowability, and cohesiveness. The incorporation of these powders significantly reduced the solubility time of milk powders, while the water activities and moisture levels of the resultant products were also within adequate extents, feasible for the safe storage of these milk powders. In another investigation, an Indigenous sub-continental sweet dairy product, Peda, was also prepared, analyzed, and finally optimized (2%) utilizing ginger powder [96]. The inclusion of ginger powder into Peda not only increased the total solid, total sugar, and ash contents, but also improved its shelf life by reducing the total plate count. 

Apart from ginger powder, the effect of ginger juice on the physicochemical and hedonic attributes of yogurt was also investigated at different concentrations. An inverse relationship was observed among the amount of ginger juice, the viability of the starter culture, and the hardness of the product. Based on the finding of this study and the sensorial characteristics of the product, the ginger juice addition at the rate of 4% (*v*/*v*) was regarded as the suitable concentration in yogurt production [97], while Allam et al. [98] reported that ginger juice (2%) incorporation into fermented milk products reduced the coagulation time and accelerated the syneresis process.

Jadhav et al. [99] formulated a functional milkshake product via the inclusion of ginger juice at four different concentrations from 2.5% to 10% (*v*/*v*). The increase in the ginger juice concentration was inversely proportional to the fat, protein, and total solid contents of the product. Considering the sensorial properties and other attributes, a 2.5% ginger juice addition in the milkshake was regarded as an optimum value. Gaur et al. [100] also successfully developed an herbal milk product by optimizing the concentrations of herbal ingredients such as ginger, turmeric, and tulsi (holy basil). 

Several studies were also designed for the potential exploitation of ginger into an herbal ice cream product. Herbal ice cream could be manufactured employing 4% ginger juice (*w*/*w*) with a better hedonic response than all other treatments including the control. Ginger juice improved the product’s resistance against melting but lowered the fat and total solid contents in the herbal ice cream [101]. Similar findings were also reported in another investigation [102]. The incorporation of ginger juice has a pertinent effect on the thermal properties but no substantial influence on the freezing characteristics of ice cream [103]. Mule et al. [104] efficiently employed ginger juice, aloe vera juice, and *L. acidophilus*-based probiotic culture to produce probiotic ice cream at the optimized levels of 3%, 6%, and 7% (*w*/*w*), respectively.

The fortification of ginger extract into fermented dairy products is another approach to bestow the favorable characteristics of ginger to the products. Ginger extract, at an 8% concentration of the low-fat yogurt formulation, not only boosted the phenolic contents and antioxidant activities, but also played a vital role in the retention of these properties [105]. The addition of ginger extract also lowers the syneresis process in the yogurt. During the storage period, there was not any remarkable effect on the acidity, but the viscosity of the product was decreased with the incorporation of ginger extract. 

In two other investigations, herbal yogurt was developed using ginger extract and evaluated for its antioxidant capacity and sensorial properties. Ginger was regarded as a better source of antioxidant properties than beet root, while the herbal yogurt containing ginger extract (2%) and goat milk was characterized with the highest antioxidant activity than other milk sources (buffalo and cow). A sensorial analysis revealed that herbal yogurt augmented with ginger extract (1%) was superior in color and appearance, while garlic extract (0.2%) was responsible for a better taste and flavor [106,107].

Ginger extract in combination with gum arabic has also been exploited in the manufacturing of a yogurt-based traditional Iranian drink, Doogh [108]. The synbiotic dairy product with maximal overall acceptability and advanced probiotic status was developed with a blend of ginger and gum arabic at the concentrations of 0.25% and 0.5%, respectively. In another attempt to produce synbiotic labneh through the efficient utilization of ginger and probiotics, an effective inhibitory effect on some food-borne pathogens was detected along with an enhanced phenolic profile and superior overall acceptability of the end product [109].

The ethical, economical, and supply chain aspects of the calf rennet compelled dairy experts to explore other potential sources of milk coagulation. Ginger and its derivatives were also analyzed as potential coagulants in cheese processing. Ginger crude extract emerged as an auspicious coagulant in soft un-ripened cheese manufactured from camel milk [110]. Ginger extract did not perform as good as commercial camel chymosin but rendered a crucial task in the value addition of the product. Further studies were also proposed to explore the coagulation capability of pure coagulation enzymes from ginger. Peshawari cheese, a traditional cheese in the Khyber Pakhtunkhwa province of Pakistan, was prepared utilizing ginger protease compared to calf rennet [111]. In a comparison between ginger protease and calf rennet, there was not any pertinent difference observed in terms of the physicochemical and microbiological properties of the Peshawari cheese. In fact, ginger protease accelerated the proteolysis and improved the organoleptic characteristics of the cheese. 

Ginger extract has been productively employed in soft cheese manufacturing as a functional ingredient. Ginger extract addition promoted the quality attributes of soft cheese by reducing the pH, oxidative rancidity, and microbial load while increasing the proteolysis, cohesiveness, and smoothness of the product. Ginger extract-fortified cheese also earned higher scores for texture, flavor, and overall acceptability than the control. Ginger extract (4%) coupled with the pickling of the soft cheese was reported with superior quality characteristics [112]. 

Herbal ice cream with health-promoting attributes and a reduced sugar content could also be prepared by formulating ginger extract and xylitol [113]. The physical characteristics of the product were not altered significantly by incorporating herbal ingredients, but the antioxidant activity and phenolic contents were improved, and an alluring appearance was imparted to the final product. Another study was also conducted to characterize the volatile compounds of mature ginger rhizome and its potential utilization to develop a functional ice cream product [114]. The maturity of ginger rhizome exhibited a direct and inverse relationship with zingiberene and ar-curcumin, respectively. A value-added functional ice cream with enviable sensorial characteristics and innovative ginger flavoring could be produced by incorporating the gingerols from freeze-dried ginger extract.

In another study, different forms of ginger such as ginger juice, paste, candy, and powder were formulated into ice cream to analyze its physicochemical and hedonic attributes. The addition of ginger juice and paste decreased the fat, total solid, and overrun contents, while the incorporation of ginger candy and powder increased the fat, total solid, and overrun contents, but the phenolic contents and antioxidant capacity were increased in all inclusions. The product’s resistance to melting improved in all preparations of the ginger, and the optimized incorporation level of all ginger forms were 4% paste, 6% juice, 10% candy, and 1% powder [115]. 

A novel approach of utilizing ginger essential oil in yogurt preparation was also successfully applied to evaluate the quality characteristics of the final product. Ginger essential oil enrichment (1000 ppm) lowered the syneresis and pH, while it enhanced the shelf life of the product and improved the overall acceptance of the product [116]. Efforts were also made for the efficient employment of essential oils from ginger, thyme, and clove in soft cheese formulation [117]. The inhibitory effects of these essential oils were reported against different food-borne pathogens, and the sensorial attributes were also improved with the addition of these essential oils.

**Table 2:** Some important investigations on the incorporation of ginger and its derivatives in dairy products.

Derivative	Product	Positive Effect	Reference
Ginger Powder	Yogurt	↑ Antioxidant Activity; Phenolic Contents; Total Solids; Viscosity; Textural Properties, Storage Stability; ↓ Syneresis; pH; Time; Energy; ≈ Sensorial Characteristics	[92,93]
	PEDA	↑ Functionality; Shelf Life; ↓ Microbial Growth; Cost	[96]
Ginger Juice	Yogurt	↑ Total Phenolic Contents; Antioxidant Total Solids; Viscosity; ↓ Coagulation Time; pH	[97,98]
	Ice Cream	↑ Antioxidant Activity; Phenolic Content; Sensorial Characteristics; Melting Resistance	[101,102,104]
Ginger Extract	Yogurt	↑ Antioxidants; Phenolic Contents; Sensorial Properties; ↓ Syneresis; pH	[105,107]
	Labneh	↑ Antimicrobial Activity; Antioxidants; Phenolic Contents; Sensorial Characteristics; ↓ Microbial Growth; pH	[109]
	Cheese	↑ Antioxidant Activity; Total Phenols; Oxidative Stability; Storage Stability; Sensorial Characteristics; Coagulation ↓ Microbial Growth	[110,112]
	Ice Cream	↑ Antioxidants; Total Phenolic Contents; Sensorial Properties; ↓ Microbial Count; ≈ Viscosity; Overrun	[113,114]
Ginger Protease	Cheese	↑ Proteolysis; Functionality; Coagulation; Sensorial Properties	[111]
Ginger Essential Oil	Soft Cheese	↑ Antioxidants; Phenolic Contents; Sensorial Characteristics; ↓ Microbial Growth	[117]

↑ = Increased/Improved, ↓ = Lower/Decreased, ≈ = Negligible/Unchanged.

In conclusion, ginger’s diversified applications in dairy products have demonstrated its vast potential as a functional ingredient. Among the different forms of ginger, ginger extract came up as an extensively employed form, followed by ginger juice, powder, and essential oil. Ginger extract exhibited its aptitude for improving the overall techno-functional characteristics of dairy products and offering persuasive coagulation properties for cheese manufacturing. While the use of all forms of ginger proved to be effective in elevating the functionality and efficacy of dairy products, at the same time, some quality and hedonic characteristics were also compromised, but the incorporation of ginger extract was identified with higher functional and physicochemical improvements and minimal quality defects.

### 5.3. Beverages

Like other food sectors, the incorporation of functional food ingredients including ginger has also been investigated in beverages (Table 3). In the manufacturing of a ready-to-drink ginger–cocoa beverage, ginger powder and cocoa were optimized at an 8% concentration with low-fat cocoa powder, respectively, as higher concentrations of ginger and fat interfere with two decisive quality factors, sedimentation and viscosity [118]. A successful attempt was also made to develop a ready-to-drink and ready-to-reconstitute ginger-based beverage with superior functionality and a 6-month shelf life at an ambient temperature [119]. 

Ginger extract in combination with lemon juice rendered an excellent job as a natural preservative in a carrot–mandarin RTS drink. During storage, a few modifications were observed in the physicochemical properties, but the product characteristics remained consistent to a large extent [120]. A ginger and Indian gooseberry-based herbal beverage was also prepared and investigated by replacing sugar with aspartame, an artificial sweetener [121]. Liu et al. [122] recommended atmospheric pressure sterilization for a ginger-flavored beverage due to its feasibility and capability to retain the important volatile compounds.

Fresh ginger extract was adequately exploited in the production of a ginger-based functional carbonated beverage. The functionality and sensorial characteristics of the drink were improved with the addition of ginger rhizome extract, but the shelf life was largely associated with the carbonation level of the product [123]. In another study, supercritical extraction was mentioned as a superior extraction approach among conventional and ultrasonic extraction techniques, while mint was detected with marginally better phenolic and antioxidant profiles than ginger. A ginger–mint drink of high nutraceutical value was prepared at 0.4% concentrations of each extract with favorable physicochemical and hedonic characteristics [124]. 

Ginger is also a vital ingredient in Zobo drink, a traditional and indigenous Nigerian drink prepared from *Hibiscus sabdariffa* flowers [125]. The fortification of Zobo drink with ginger and garlic extracts substantially reduced the microbial load. The degradation of sensorial features with storage was observed in the control samples, while the samples that incorporated ginger and garlic not only had improved sensorial attributes but also maintained these characteristics during storage [126]. 

Ginger has also been formulated in various functional beverages in the form of blends with different spices, herbs, fruits, and other food ingredients. A soybean, ginger, and mango-based functional drink was developed to effectively exploit the nutritional and functional values of these ingredients, which were consequently attributed with feasible sensorial properties and overall acceptability [127]. An optimum formulation (*v*/*v*) was furnished for a functional beverage utilizing ginger extract (15.1%), tamarind extract (9.9%), turmeric extract (5.0%), sugar solution (40%), and water (30%). 

Ginger extract, Kencur extract, and their combination blend were incorporated into an instant temulawak beverage, an indigenous Indonesian drink, to evaluate their impacts on the physicochemical and sensorial attributes of the final product. The inclusion of ginger extract in a concentration of up to 15% enhanced the flavor, aroma, and overall acceptance of the Temulawak drink [128]. Another study associated ginger extract with an improvement in the sensorial properties, but there was no significant advancement in the antioxidant activity with the ginger addition to a cocoa, ginger, and hibiscus flower-based beverage product [129]. A ginger-based beverage with hyperthermic activity was also found to be efficient against peripheral skin extremities in women experiencing mild cold sensitivity [130]. 

The integration of two different food ingredients could ameliorate the defective quality attributes of the intended food product. The fusion of a better coloring feature from tea and a favorable flavoring attribute from ginger resulted in a superior acceptable beverage product [131]. During an antioxidant evaluation in tea-ginger extract, the powder concentration was reported as the most influential process parameter than the time and temperature [132]. The supercritical ginger extract (0.3%) was preferred over the conventional ginger extract in iced ginger tea and functional coffee substitute production due to its exceptional performance, safety, purity, and overall acceptability [133,134]. Ginger extract (10%) was formulated and optimized with non-decaffeinated Robusta coffee (40%) with an improved functional value and without any adverse effects on the sensorial and quality characteristics [135].

**Table 3:** A few important studies on the addition of ginger and its derivatives to beverage products.

Derivative	Product	Positive Effect	Reference
Ginger Powder	Cocoa Beverage	↑ Total Phenolic Contents; Antioxidant Activity; Viscosity	[118]
Ginger Extract	Carrot and Kinnow RTS Drink	↑ Antioxidant Activity; Preservation; ↓ Microbial Growth; ≈ Sensorial Properties	[120]
	Zobo Drink	↑ Functionality; Shelf Life; Sensorial Properties; ↓ Microbial Count	[126]
	Black Tea	↑ Antioxidant Activity; Phenolic Contents; Sensorial Properties	[131]
	Ginger Iced Tea	↑ Nutraceutical Compounds; Shelf Life; Organoleptic Attributes	[133]
	Robusta Coffee	↑ Antioxidant Activity; Phenolic Contents; ≈ Sensorial Properties	[135]

↑ = Increased/Improved, ↓ = Lower/Decreased, ≈ = Negligible/Unchanged.

The above-mentioned research findings have provided insight into the exploitation of ginger in beverage products. Even in beverages, the employment of ginger extract seems to dominate over the rest of the ginger derivatives due to its better assimilation and conducive characteristics. The incorporation of ginger into different drinks not only augmented but also maintained the shelf life, nutraceutical value, and antimicrobial activity. 

### 5.4. Meat and Fish Products

Several investigations and experiments were also conducted to figure out the effects of ginger and its derivatives in various forms on meat and fish products (Table 4). Rabbit meat burger patties were fortified with ginger powder at two different concentrations (1% and 2%) and significantly improvised the fatty acid profile by accelerating the polyunsaturated fatty acids [136]. Ginger powder also increased the antioxidant capacity and reduced the lipid peroxidation, subsequently catering a meat product with a better shelf life and enriched nutritional status. Mancini et al. [137] also designed an experiment to enhance the quality of pork burgers through ginger powder incorporation. The physiochemical attributes, antioxidant capacity, fatty acid profile, microbial growth, and lipid oxidation of the ginger powder-enriched pork burger were evaluated, and the results were in agreement with the previous study. In another investigation, ginger was designated as a potential alternative of artificial antioxidants in pasteurized canned meat products [138]. Ginger and papain powders were also competently exploited as natural tenderizing agents in buffalo calf meat [139].

A comparative analysis was conducted to assess the effects of three different spices as potential organic preservatives in raw chicken meat products. Clove powder (0.2%), even in a very small quantity, compared to ginger (3%) and garlic (2%) pastes performed very well to improve the oxidative stability, physicochemical properties, and antimicrobial activities in raw chicken meat with refrigerated storage [140]. Ginger and pineapple juices had also been examined as promising marinating agents in raw and cooked goat meat [141]. Ginger juice not only provided oxidative stability but also developed the hedonic features by according a bright red color and strong marinating odor and reducing the gamey flavor of Saanen goat meat. In another investigation, papain juice was described as a better tenderizing agent than ginger and pineapple juices as it induced a mild disruption in the muscles of goose meat [142]. Fermented ginger paste was characterized along 25 metabolites with substantial antimicrobial activity and its application on chicken meat efficiently enhanced the shelf life through a significant reduction in the microbial load [143]. Similar findings were also reported by Abdel-Naeem and Mohamed [144] regarding the application of ginger and papain extracts in camel meat patties. Sukada et al. [145] stipulated that pineapple extract had superior hedonic characteristics than papaya leaf and ginger extracts when practiced on Bali beef through the immersion method. 

It was revealed that the competence level of ginger was as good as sodium ascorbate to combat oxidative rancidity, while the textural and sensorial properties of meat products with ginger were better than synthetic antioxidants [146]. Ginger extract also performed efficiently in the case of smoked buffalo meat as the microbial, physicochemical, and sensorial attributes did not exhibit any remarkable changes during the storage period. Ginger extract not only regulated and maintained the microbial count within certain limits but also enhanced the functionality of the cooked meat products.

Smoked mackerel fish was treated with ginger extract to evaluate its influence on the shelf life and hedonic characteristics [147]. Ginger extract (5%) significantly improved the stability and functional value of the smoked fish through its antimicrobial and antioxidative features. The sensorial quality of fish with the incorporation of ginger extract was also better and more persistent than other treatments even during storage. Mattje et al. [148] disclosed that the antioxidant activity of supercritical ginger extract is greater than ginger essential oil, which, consequently, was detected in the treatment of tilapia fish burgers with ginger. Supercritical ginger extract also enhanced the shelf life of fish burgers from 6 to 8 days, but it disturbed the overall perception due its unfavorable spicy flavor.

A variety of synthetic tenderizers and proteolytic enzymes had been engaged in the tenderization of meat. Currently, natural tenderizers are in demand due to certain constraints of synthetic tenderizers such as safety issues, unpleasant tastes, and over-tenderization. Ginger extract (5%) was recommended as a potential tenderizer for Yak meat as it enhanced the cooking yield, tenderness, and lipid oxidative stability during storage [149,150]. 

Researchers had also adopted a distinct approach to improve the meat quality through feeding ginger, as a potential dietary supplement, to the animals. Ginger and its derivatives in various forms were highly effective in inducing favorable characteristics such as lowering the cholesterol and improving the egg functionality and carcass quality in poultry animals [151,152]. The dietary augmentation of rabbits was also carried out through ginger powder supplementation. This augmentation improved the resistance to lipid oxidation without causing any intervention to the productive functions of the rabbits [153]. The administration of red ginger as an herbal–biotic feed ingredient (1.5%) competently boosted the productive activity and carcass quality in broilers [154]. Ginger extract loaded with gingerol was effectively employed to derive its health-promoting features for the prosperity of heat-stressed broilers [155]. Healthy and good-quality meat could be produced through catering ginger and rosemary essential oils to New Zealand white (NZW) rabbits. The provision of both essential oils (0.5%) promoted the feed exploitation, growth efficiency, and meat quality in growing rabbits [156]. Meanwhile, essential oils from different spices and herbs including ginger were exploited for their potential applications as natural preservatives to improve the oxidative stability in meat and meat products [157]. 

**Table 4:** Some important studies on the addition of ginger and its derivatives to meat and fish products.

Derivative	Product	Positive Effect	Reference
Ginger Powder	Rabbit Burgers	↑ Antioxidant Activity; Fatty Acid Profile; Shelf Life; ↓ Lipid Peroxidation	[136]
	Pork Burger	↑ Antioxidant Activity; PUFA; Oxidative Stability; Sensorial Characteristics; ↓ Microbial Load	[137]
Shredded Ginger	Canned Pork	↑ Antioxidant Activity; Tenderness; Organoleptic Properties; ↓ Microbial Load	[138]
Ginger Paste	Chicken Meat	↑ Antioxidants; Antimicrobial Activity; Oxidative Stability ↓ Microbial Load	[140,143]
Ginger Juice	Goat Meat	↑ Antioxidant Activity; Tenderness; Oxidative Resistance; Storage Stability; Overall Acceptability; ↓ Gamey Odor	[141]
	Beef	↑ Antioxidant Activity; Tenderness; Sensorial Properties; ↓ Cooking Loss	[145]
Ginger Extract	Camel Meat Patties	↑ Antioxidants; Lipid Stability; Collagen Solubility; Sensorial Properties	[144]
	Smoked Buffalo Meat	↑ Antioxidant Activity; Tenderness; Sensorial Characteristics; ↓ Cooking Loss; Microbial Load	[146]
	Smoked Fish	↑ Antioxidants; Shelf Life; Organoleptic Attributes; ↓ Mold Count; Oxidative Rancidity	[147]
	Tilapia Fish Burger	↑ Antioxidant Activity; Shelf Life; ↓ Lipid Oxidation	[148]
	Yak Meat	↑ Antioxidant Activity; Tenderness; Oxidative Resistance; Cooking Yield; ↓ Microbial Load	[150]
Gingerol Rich Extract	Chicken Feed	↑ Antioxidant Activity; Bioactive Compounds; Meat Quality	[155]

↑ = Increased/Improved, ↓ = Lower/Decreased

As per the aforementioned studies, ginger has an extensive application potential in the meat industry, embellishing meat products with an array of favorable characteristics. The incorporation of ginger into meat products competently ameliorated the nutritional value, oxidative stability, lipid profile, and antimicrobial activity of the meat products. The ability of ginger to provide elegant antioxidant and tenderization activities without compromising the sensorial and textural attributes of meat products has also provided another priority lead over artificial antioxidants and tenderizing agents. The inclusion of ginger in the diets of animals as a dietary supplement has also emerged as a novel and efficient approach, which remarkably improves the fatty acid profile and carcass quality of the animals.

### 5.5. Film, Coating, and Packaging

Ginger and its bioactive compounds have also been examined for their potential applications as edible coatings, films, or active ingredients in packaging (Table 5). The use of glycerol as a green solvent was also practiced to extract the bioactives from ginger powder [159]. The resultant glycerol ginger extract (GGE) was incorporated in a chitosan-based edible coating for walnut kernels. This coating formulation was effective against the lipid oxidation and fungal spoilage of walnuts under storage conditions. Another study disclosed that the combination of ginger and garlic is a more efficient preservative approach than other famous preservatives such as BHT and Nisin [160]. The application of ginger–garlic extract in the vacuum packaging of Herring fish fillets increases its stability against oxidation and microbial spoilage and also imparts favorable sensorial attributes to the product.

Whey protein isolate (WPI) coating on Kashar cheese was characterized with water and fat barrier attributes, which were significantly developed with the addition of 1.5% (*v*/*v*) ginger essential oil, regarded as whey protein isolate ginger (WPIG) coating [161]. WPIG coating was also found to be more competent in the shelf life extension of Kashar cheese due to the anti-bacterial activity of ginger essential oil against *E. coli* and *S. aureus*, while Tsironi et al. [162] also expressed that WPI films enriched with 1% ginger essential oil efficiently prevented the microbial spoilage of minced lamb meat with acceptable sensorial properties until 8 days of refrigerated storage. 

Sodium alginate/agar (SA) coating enhanced the shelf life and maintained the quality attributes of beef; moreover, these characteristics were substantially improved with the incorporation of ginger essential oil (SAG) into edible coating [163]. Compared to the control, these coating formulations, SA and SAG, extended the shelf life of beef at a chilling temperature by 3 to 6 days and 9 days, respectively, through their counter actions against lipid oxidation and microbial decay. Ginger essential oil- fortified (6%) sodium caseinate-based edible coating fabricated through nanoemulsion efficiently improved the shelf life of raw chicken meat [164]. This edible coating significantly enhanced the microbial resistance and embellished the sensorial characteristics of the chicken meat, but no remarkable increment in antioxidant activity was observed. Researchers also developed a peculiar coating formula based on apple peel extract and zein loaded with ginger essential oil to prolong the shelf life of raw chicken meat [165]. 

Fish sarcoplasmic protein and chitosan composite films enriched with ginger essential oil (up to 3%) were analyzed for their physicochemical and antioxidant features [166]. Ginger essential oil (GEO) nanoemulsion furnished the composite films with better elongation at break, antioxidant, and water barrier properties, while on the other hand, GEO was also liable to accord the inferior thermal and mechanical properties. In another investigation, a blend containing equal concentrations of ginger and cinnamon essential oils was incorporated into chitosan films for its potential utilization in pork packaging. These composite films were reported with better thickness and opacity, restricted the lipid oxidation and microbial spoilage, and enhanced the antioxidant activity in pork slices stored at a refrigeration temperature [167]. The two different studies also evaluated the effects of ginger and cinnamon essential oil (EO) supplementation on soy protein isolate (SPI) and sodium caseinate-based edible films [168,169]. The water vapor permeability was almost unchanged with both EOs, while the optical features were significantly affected by the cinnamon essential oil. In SPI films, cinnamon EO was more resistant and stretchable than ginger EO, while both EOs were incompetent to cater any improvements in the lipid oxidation stability in sodium caseinate film.

Zhang et al. [170] also formulated a Tilapia fish skin gelatin, ginger EO, and ZnO nanoparticle-based microemulsion nanofilm for the shelf life extension of fresh meat where ginger EO was responsible for better mechanical characteristics and improved functionality status with elevated antioxidant and antimicrobial properties. Researchers have also struggled to employ ginger and its bioactive compounds in active packaging to prolong the shelf life of food products. Chitosan-based bio-nanocomposite enriched with ginger EO (1%) was effectively exploited to extend the shelf life of poultry without any compromise on the sensorial properties [171].

It is evident from the foregoing literature that ginger oleoresins have promising applications in edible films, coatings, and the packaging sector, where ginger essential oil has better and extensive utilizations than ginger extract. The consolidation of ginger significantly improved the oxidative stabilities, antimicrobial activities, and sensorial properties of the intended food products. Ginger also performed better than the synthetic preservatives, improved the shelf life, and retained the quality attributes of the products.

**Table 5:** Some important studies on the application of ginger and its derivatives to films, coatings, and packaging.

Derivative	Product	Positive Effects	Reference
Ginger Extract	Chitosan-Based Edible Coating (Walnut)	↑ Antioxidant Activity; Shelf Life; Oxidative Stability; ↓ Fungal Spoilage	[159]
	Garlic Extract cum Vacuum Packaging (Fish Fillet)	↑ Antioxidant Activity; Shelf Life; Oxidative Stability; Sensorial Properties; ↓ Microbial Spoilage	[160]
Ginger Essential Oil	Whey Protein Isolate Coating (Cheese)	↑ Anti-bacterial Activity; Shelf Life; Water and Fat Barrier Properties	[161]
	Edible Coating (Chicken Meat)	↑ Shelf Life; Sensorial Characteristics ↓ Microbial Growth; ≈ Antioxidant Activity	[164]
	Fish Protein and Chitosan Composite Films	↑ Antioxidant Activity; Shelf Life; Water Barrier Properties	[166]
	Cinnamon EO Chitosan Films (Pork)	↑ Antioxidant Activity; Shelf Life; Film Thickness; ↓ Microbial Spoilage; Lipid Oxidation	[167]

↑ = Increased/Improved, ↓ = Lower/Decreased, ≈ = Negligible/Unchanged.

## 6. Conclusions and Future Prospectus

Ginger, with strong antioxidant, antimicrobial, and other functional activities, has a great potential for employment in the food industry. In this review, many studies have been presented regarding the acquirement of the bioactive compounds of ginger in different forms such as powder, extract, juice, paste, and essential oil. The application of ginger and its derivatives in a variety of food products has been extensively investigated and reported with auspicious results. Among all of the derivatives of ginger, ginger extract was found to be a more practicable, efficient, and versatile form of ginger for food applications. Ginger essential oil is another efficient variant of ginger, but its food applications are limited because of its strong aroma and immiscibility with certain food products.

These studies have also revealed another positive aspect of ginger, which is that it could be employed in a wide range of food products, covering almost all leading sectors of the food industry. The incorporation of ginger, in its various forms, thoroughly improved the functionality and bioactivity of the intended food products, but the physicochemical and sensory properties of these products were also compromised in a legitimate number of studies. 

Further studies mainly focused on the efficacy, optimization, and assimilation of ginger bioactives into food products without compromising the physicochemical characteristics and sensory profiles of the products must be carried out. The probable synergistic effect of ginger bioactives with other compounds and toxicity studies of different compounds in their purest forms should also be investigated to ensure the safe use of these functional compounds. 

## Data Availability

Not applicable.
